# Hydroxychloroquine in children with proliferative lupus nephritis: a randomized clinical trial

**DOI:** 10.1007/s00431-023-04837-0

**Published:** 2023-02-08

**Authors:** Fatma Sayed Gheet, Heba El-Sayed Dawoud, Waleed Ahmed El-Shahaby, Shymaa Mohamed Elrifaey, Hend Hassan Abdelnabi

**Affiliations:** Pediatric Department, Faculty of Medicine, Tanta University, El-Geesh Street, Tanta, 31527 Egypt

**Keywords:** Children, Lupus nephritis, Hydroxychloroquine, Efficacy, Side effects

## Abstract

Hydroxychloroquine (HCQ) is an antimalarial agent used to treat mucocutaneous, musculoskeletal, constitutional manifestations of systemic lupus erythematosus (SLE). This study assessed the efficacy and side effects of HCQ in children with proliferative lupus nephritis (LN). This double-blind, randomized, placebo-controlled trial study was conducted on 60 children with proliferative LN classes III and IV treated with steroids and a mycophenolate (MMF) regimen. Patients were categorized into two groups, the HCQ group (*n* = 30) and the placebo group (*n* = 30). They were evaluated initially at 6- and a 12-month follow-up by mucocutaneous, ophthalmological examination, and investigations (BUN, creatinine, 24 h proteinuria, triglycerides (TG), cholesterol, Antids-DNA, C3, C4). Disease activity was assessed using the SLE disease activity index (SLEDAI-2 k). After 12 months, TG, cholesterol, 24 h proteinuria, Antids-DNA, and SLEDAI score were significantly decreased in the HCQ group (*P*: 0.002, 0.012, 0.031, 0.001, respectively). After 12 months, the cumulative probabilities of developing primary end-points (LN partial and complete remission) were 40% and 60% in the HCQ group versus 53.3% and 36.7% in the placebo group (*P*: 0.002). After 12 months, the HCQ group experienced mucocutaneous alopecia (3.3%), hyperpigmentation (10%), and ophthalmological mild retinal changes (6.7%), but they did not differ significantly from the placebo group.

*  Cunclusion*: HCQ improved the disease and LN activity in children with proliferative LN, with documented skin hyperpigmentation and mild retinal changes following HCQ use in a few cases. This study was registered on http://www.clinicaltrials.gov/ with trial registration number (TRN): NCT03687905, September 2018 “retrospectively registered.”

**Table:** 

**What is Known:**
• *Hydroxychloroquine (HCQ) is documented as an adjunctive treatment in children with systemic lupus erythematosus (c-SLE) LN with efficacy in improving lupus musculoskeletal and mucocutaneous manifestations*.
• *Due to the paucity of studies, its effects and side effects in children with LN remain unclear*.
**What is New:**
• *This pilot randomized clinical trial assessed the efficacy and adverse effects of HCQ in children with proliferative LN*.
• *HCQ had numerous advantages for LN, including rapid and sustained remission, antilipidemic effect, and rapid improvement of kidney functions*.

**Supplementary Information:**

The online version contains supplementary material available at 10.1007/s00431-023-04837-0.

## Introduction

LN occurs in about 60% of children with systemic lupus erythematosus (c-SLE), ranging from silent disease to severe renal insufficiency. Despite advances in therapy, morbidity and mortality of LN remain high, leading to kidney failure in 17–25% of patients [1, 2]. All guidelines recommend a renal biopsy when a patient with SLE has proteinuria (> 500 mg/day or > 3 + on urine dipstick) to confirm the diagnosis, assess disease activity or chronicity, and guide treatment. LN is classified histologically according to the International Society of Nephrology/Renal Pathology Society (ISN/RPS) into sixth classes, Class I: Minimal mesangial LN, Class II: Mesangial LN, Class III: Focal proliferative LN, Class IV: Diffuse proliferative LN, Class V: Membranous LN, Class VI: Advanced sclerosis LN [3, 4]. HCQ is an antimalarial drug used to treat mucocutaneous, musculoskeletal, and constitutional manifestations of SLE, with photoprotective, dyslipidemic, and antithrombotic actions [5]. HCQ has side effects occurring with long-term use and high cumulative doses, such as retinopathy, corneal deposits, maculopathy, decreased visual acuity, impaired night vision, hyperpigmentation, alopecia, skin rash, cardiomyopathy, ototoxicity, myopathy, leukemia, and hemolysis [6]. HCQ effects in LN treatment are still unclear, with a paucity of studies on children, so we aimed to evaluate its efficacy and side effects in treating children and adolescents with proliferative LN.

## Materials and methods

### Study design

This double-blind randomized-controlled trial was conducted on 60 children (sample size was calculated using G*Power 3.1.9 with effect size 0.15, *α* error 0.05 and power 95%) at the Pediatric Department, Tanta University Hospitals, Egypt, between March 2019 and December 2021. Inclusion criteria were children aged ≤ 18 years who were newly diagnosed with c-SLE according to SLICC criteria with proliferative LN (LN classes III, IV, or mixed with other classes). We excluded patients with LN classes I, II, and V; end-stage kidney disease (ESKD); ophthalmologic evidence of retinopathy at initial disease presentation; neurolupus; nonadherence to treatment and follow-up; and seriously compromised patients. We used the computerized covariate adaptive randomization for patients’ selection who participated in the study. Sealed numbered containers were used for allocation concealment. This study was performed in line with the principles of the Declaration of Helsinki.

### Study approval

Study approval was granted by the Ethics Committee of Faculty of Medicine, Tanta University (No. 32490/7/18) and from Protocol Registration and System on http://www.clinicaltrials.gov/ with trial registration number (TRN): NCT03687905, September 2018; informed consents were obtained from parents and participants in this study. The trial started with 98 newly diagnosed c-SLE cases, 24 cases were excluded as they didn't have LN classes III or IV, 6 cases declined to participate, and after allocation, 9 cases were missed and 7 cases were excluded as they were not adherent to follow-up (as shown in the CONSORT follow-diagram and Table 1).
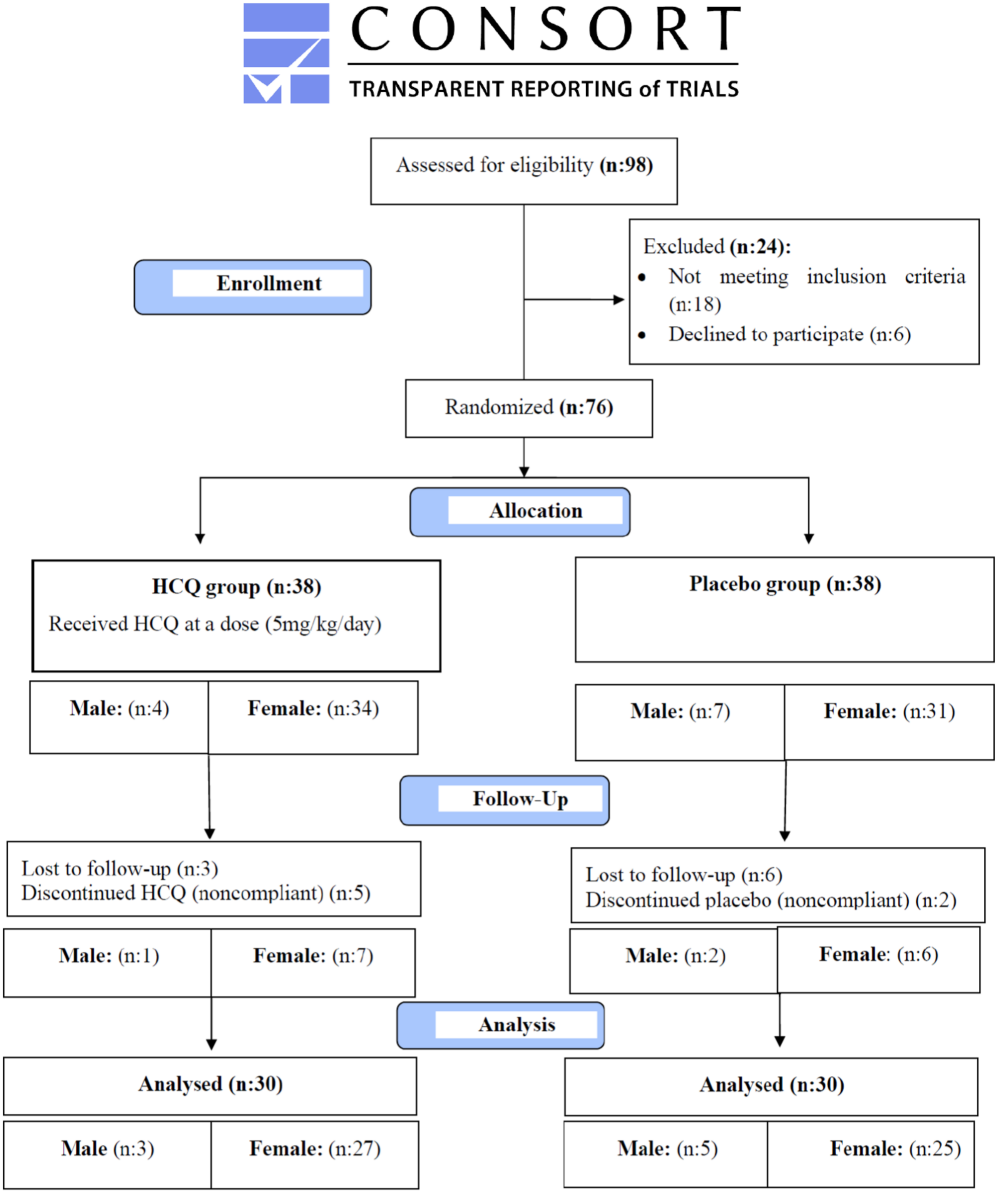

**Table 1:** Comparison between cases who completed the study and those who were lost at follow-up

**Data**		**All randomized cases (*n*: 76)**		***P***
		**Cases completed the study (*n*: 60)**	**Lost cases at follow-up (*n*: 16)**	
		Adherent to treatment and followed-up regularly	Non-adherent to treatment and did not follow-up regularly	
**Age (years)** (mean ± SD)		13.3 ± 2.2	13.7 ± 2.5	0.561
**Sex** (*n*, %)	Male	8 (13%)	3 (19%)	
	Female	52 (87%)	13 (81%)	0.167
	M/F ratio	01:6.5	01:4.3	
**Family history of rheumatological diseases** (*n*, %)		7 (12%)	0	0.001^*^
**LN classes:** (*n*, %)				
▪ LN III		32 (54%)	11 (69%)	
▪ LN IV		26 (43%)	5)31%)	0.275
▪ LN IV-V		2 (3%)	0	
**SLEDAI** (mean ± SD)		24.7 ± 3.2	23.4 ± 4.2	0.733

^∗^ means a statistically sigificant difference (P ≤ 0.05)

### Treatment regimen and follow-up

All studied c-SLE patients had proliferative LN and received steroids and MMF regimen: (a) induction phase (three daily intravenous pulse methylprednisolone doses (500 mg/m^2^/dose) was given followed with oral steroids (2 mg/kg/day), mycophenolate mofetil (MMF) (1200 mg/m^2^/day) combined with angiotensin converting enzyme (ACE) inhibitors, and hydroxychloroquine (5 mg/kg/day) for 4 week;s then, steroids were tapered to (5 mg/day) after 6 months. This regimen (low steroid dose 5 mg/day) + MMF 1200 mg/m^2^/day) was continued for another 18 months (Maintenance phase). If complete remission (proteinuria reduction < 500 mg/day, normalized serum creatinine with controlled blood pressure) was not achieved after 12 months of follow-up (resistant and refractory cases), MMF is shifted to cyclophosphamide (CYC) (500 mg/m^2^/4 weeks) for 6 doses; then, once every 3 months for another 18 months, cyclosporine A (CsA) (5 mg/kg/day) is added as a third line in addition to steroids and MMF (augmentation regimen) or rituximab is given (357 mg/m^2^/week) [7] as shown in the following diagram:
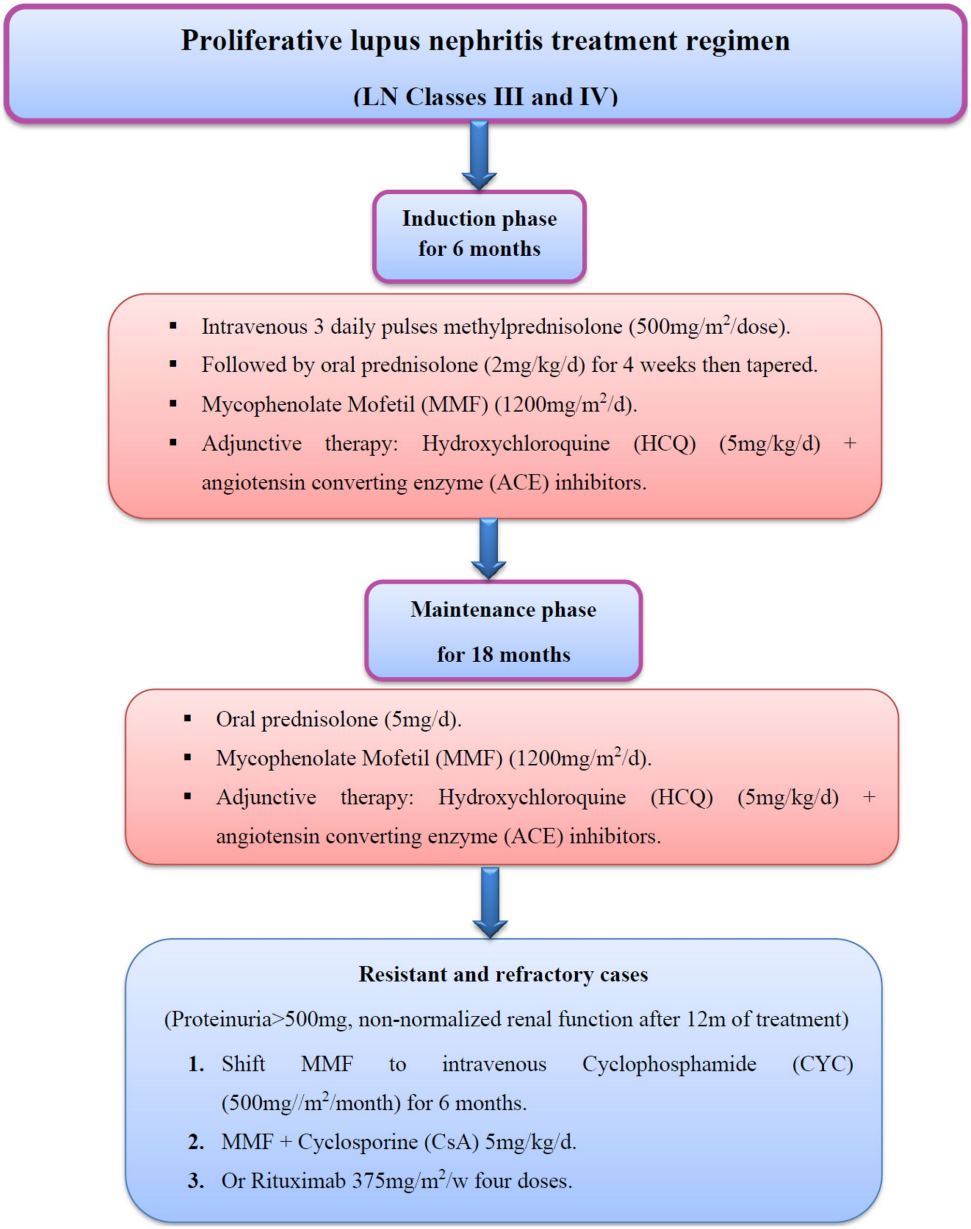


The studied patients were randomized into two groups: (1) HCQ group: Thirty patients received HCQ at a dose (5 mg/kg/day) and (2) placebo group: Thirty patients received a placebo (sugar pills like the shape and package of HCQ pills that were prepared in Tanta Faculty of Pharmacy). At initial disease presentation and throughout treatment follow-up at 6 and 12 months, all patients were subjected to the following: history taking, clinical examination: thorough general and mucocutaneous examination (malar rash, pigmentation, pruritis, eruption, alopecia, and oral ulcer), ocular examination (visual field, which is the most sensitive subjective investigation for the diagnosis of HCQ retinopathy done by confrontational visual field exam, visual acuity; using Snellen test or Random E chart and fundus examination using fundus auto-fluorescence (AF) and optical coherence tomography (OCT) for detection of any lesion in the early stage of disease). Laboratory investigations included (24 h proteinuria, blood urea nitrogen (BUN), creatinine, cholesterol, triglyceride (TG), anti-double-stranded DNA (anti-dsDNA), complements C3 and C4). Renal biopsies have been done for all cases as they met one of the following criteria: increasing serum creatinine and proteinuria of ≥ 0.5 g/24 h with hematuria or cellular casts that were evaluated according to (the ISN/RPS) grading system [8]. Assessment of the disease activity: using systemic lupus erythematosus disease activity index 2000 (SLEDAI-2 k), the disease activity was categorized as follows: no activity (SLEDAI < 4), mild activity (SLEDAI 4–7), moderate activity (SLEDAI 8–11), and severe activity (SLEDAI ≥ 12) [9].

### Treatment assessment

Primary end-points: (1) Assessment of the disease activity: no activity (SLEDAI < 4). (2) Assessment of the renal activity: Partial remission (PR) is defined as a reduction of proteinuria by at least 50% and to < 3 g/day or protein/creatinine ratio (PCR) (300 mg/mmol) and kidney function stabilization (10–15% of base line). Complete remission (CR) is defined as a reduction of proteinuria < 0.5 g/day or protein/creatinine ratio (PCR) (50 mg/mmol) and kidney function stabilization; no remission is failure to achieve a partial or complete response within 6–12 months of starting therapy [10, 11]. Secondary end-points: (1) Occurrence of disease flaring: (SLEDAI ≥ 4), (2) LN activity: (edema, increased proteinuria > 0.5 g/day, increased kidney functions), and (3) HCQ side effects: during the 12 months of follow-up.

### Statistical analysis

Statistical analysis was done by SPSS v27 (IBM©, Chicago, IL, USA). Shapiro-Wilks’s test and histograms were used to evaluate the normality of the distribution of data. Quantitative parametric data were presented as mean and standard deviation (mean ± SD) and were analyzed by *t*-test. Quantitative non-parametric data were presented as median and interquartile range (median IQR), and were analyzed by Mann–Whitney. Qualitative variables were presented as frequency and percentage (*n*, %) and were analyzed by chi-square test. Generalized estimating equations (GEE) was used to analyze longitudinal repeated measures of studied groups during the follow-up visits. Multivariate Cox regression estimate the renal survival (time of partial and complete remission). *P* value ≤ 0.05 was considered statistically significant.

## Results

**Fig. 1:**
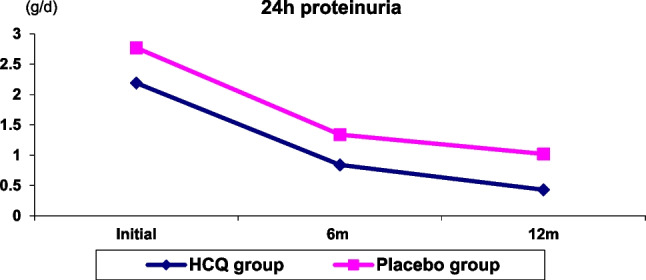
24 h proteinuria of studied groups

**Fig. 2:**
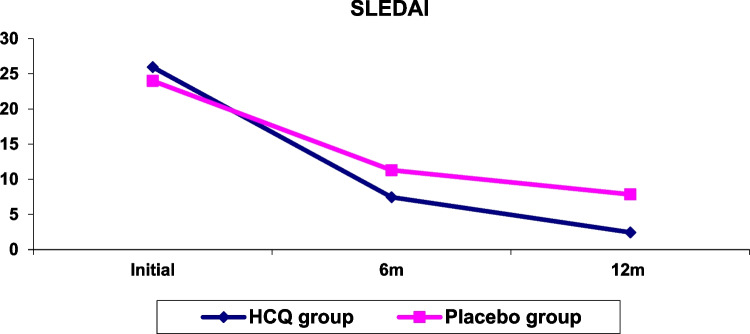
SLEDAI score of studied groups

Sixty children with proliferative LN have been included in this trial, with ages ranging between 9 and 18 years (13.3 ± 2.2), eight males (13%) and 52 females (87%), with a male-to-female ratio of (1/6.5). Thirty-two patients (54%) had LN class III, 26 (43%) had LN class IV, and two patients (3%) had mixed LN class IV-V (Table 2). The comparison between laboratory investigations of the studied groups during the follow-up durations is shown in (Table 3). As regard serum triglycerides, cholesterol, 24 h urinary proteins, and Antids-DNA levels, there were no significant differences between studied groups initially and after 6 months, while there was a significant reduction in their levels in the HCQ group after 12 months (*P*: 0.002, 0.012, 0.031, and 0.005 respectively). After 6 and 12 months, the HCQ group had a significantly lower SLEDAI score than the other groups (*P* = 0.001), with the difference being more significant after 12 months (Figs. 1 and 2). The cumulative probabilities of developing primary end-points (LN partial remission and complete remission) at 6 months of the HCQ group were 24 cases (80%) and 5 (17%), respectively, with no remission in one case (3.3%) in comparison to the placebo group that was partial remission in 20 cases (67%). There was complete remission in three cases (10%), no remission in 5 cases (17%), and relapse in 2 cases (6.7%)], at 12 m; primary end-points of the HCQ group were partial remission in 11 cases (37%), complete remission in 18 cases (60%), and relapse in 1 case (3.3%) in comparison to the placebo group that was partial remission in 13 cases (43%), complete remission in 12 cases (40%), no remission in one case (3.3%), and relapse in four cases (13%) (*P*: 0.003, 0.002). HCQ side effects documented during 1 year of follow-up are depicted in (Table 4); (1) Mucocutaneous complications such as alopecia which occurred in one case (3.3%), and hyperpigmentation, which occurred in three cases (10%), did not differ significantly from to the placebo group. (2) Ophthalmological complications: fundus examination showed mild changes in one case (3.3%) after 6 months and 2 cases (6.7%) after 12 months in the HCQ group but did not differ significantly from the placebo group, with a non-significant difference in terms of visual acuity between the two groups. Table 5 shows the multivariate Cox regression analysis of HCQ effects in studied patients (Antids-DNA (OR: 0.428, 95%CI: 0.274–0.865), TG (OR: 0.724, 95%CI: 0.534–0.924), 24 h proteinuria (OR: 0.423, 95%CI: 0.108–0.851) and SLEDAI (OR:0.352, 95%CI: 0.173–0.453)) was significant (*P*: 0.025, 0.041, 0.017 and 0.001, respectively).

**Table 2:** Demographic data of studied patients

**Data**		**All patients**	**HCQ group**	**Placebo group**	***P***
		***n*: 60**	***n*: 30**	***n*: 30**	
**Age (years)** (mean ± SD)		13.3 ± 2.2	13.3 ± 2.7	13.1 ± 2.1	0.796
**Sex** (n %)	Male	8 (13%)	3 (10%)	5 (17%)	
	Female	52 (87%)	27 (90%)	25 (83%)	0.233
	M/F ratio	01:6.5	1:9	1:5	
**Family history of rheumatological diseases** (*n*, %)		7 (12%)	3 (10%)	4 (13%)	0.677
**LN classes:** (*n*, %)					
▪ LN III		32 (54%)	15 (50%)	17 (57%)	
▪ LN IV		26 (43%)	13)43%)	13 (43%)	0.431
▪ LN IV-V		2 (3%)	2 (7%)	0	
**SLEDAI** (mean ± SD)		24.7 ± 3.2	25.9 ± 3.9	24 ± 2.8	0.081

*LN* lupus nephritis, *SLEDAI* SLE disease activity index

^*^P value: significant ≤ 0.05

**Table 3:** Comparison between laboratory investigations and SLEDAI of studied groups at initial visit, after 6 and 12 months

**Laboratory test**	**Time of follow-up**	**HCQ group**	**Placebo group**	***P***
		***n*: 30**	***n*: 30**	
**TG** (mg/dl)	Initial	230.3 ± 73.7	226.2 ± 86.4	0.166
Mean ± SD	At 6 m	124.8 ± 37.8	128.9 ± 41.5	0.815
	At 12 m	112.1 ± 50.2	168.7 ± 90.5	0.002*
	**P1**	0.035*		
**Cholesterol** (mg/dl)	Initial	286.3 ± 82.2	251.3 ± 52.9	0.117
Mean ± SD	At 6 m	199.4 ± 47.4	181.5 ± 44.9	0.886
	At 12 m	176.1 ± 39.8	215.9 ± 83.5	0.012*
	**P1**	0.041*		
**BUN** (mg/dl)	Initial	23 (19.3–65)	33.5 (15.5–64.8)	0.853
Median (IQR)	At 6 m	20 (13.8–46.3)	27 (16.3–55.8)	0.131
	At 12 m	19 (14.5–26.8)	25.5 (16.5–37)	0.667
	**P1**	0.792		
**Creatinine** (mg/dl)	Initial	1.2 (0.7–1.8)	1.21 (0.7–1.4)	0.183
Median (IQR)	At 6 m	0.7 (0.5–1)	0.8 (0.7–1.2)	0.257
	At 12 m	0.6 (0.8–1)	0.7 (0.6–0.9)	0.108
	**P1**	0.201		
**24 h proteinuria** (g/d)	Initial	2.2 (1.3–4.3)	2.8 (1.4–5.1)	0.115
Median (IQR)	At 6 m	0.8 (0.7–1)	1.34 (1.1–2.4)	0.281
	At 12 m	0.4 (0.4–0.6)	1 (0.9–1.2)	0.031*
	**P1**	0.032*		
**Serum albumin**	Initial	0.9 ± 3.1	0.7 ± 3.1	0.919
(g/dl) Mean ± SD	At 6 m	0.5 ± 3.9	0.5 ± 3.6	0.637
	At 12 m	0.7 ± 4.3	0.7 ± 4	0.188
	**P1**	0.82		
**Anti-ds DNA** (u/ml)	Initial	373.5 ± 142.4	362.7 ± 179.7	0.833
Mean ± SD	At 6 m	106 ± 45.1	110.6 ± 41.8	0.547
	At 12 m	48 ± 15.3	93.8 ± 36.7	0.005*
	**P1**	0.712		
**C3** (mg/dl)	Initial	74.5 ± 33.6	76.6 ± 30.3	0.837
Mean ± SD	At 6 m	92.7 ± 25.8	96.4 ± 33.9	0.696
	At 12 m	95.5 ± 39.1	81 ± 43.6	0.275
	**P1**	0.591		
**C4** (mg/dl)	Initial	11.3 ± 6.2	13.2 ± 5.3	0.416
Mean ± SD	At 6 m	20.6 ± 7.9	20.1 ± 7.6	0.273
	At 12 m	21.3 ± 7.8	19.6 ± 8.7	0.636
	**P1**	0.364		
**SLEDAI**	Initial	26 ± 4	24 ± 2.9	0.081
Mean ± SD	At 6 m	7.5 ± 1	11.3 ± 1	0.001*
	At 12 m	2.5 ± 0.5	7.9 ± 1.2	0.001*
	**P1**	0.001*		

*TG* triglyceride, *BUN* blood urea nitrogen, *Anti-ds DNA* anti-double stranded DNA antibody, *C3* complement 3, *C4* complement 4, *SLEDAI* SLE disease activity index, *P* significance of *t* test, *P1* significance of generalized estimating equations (GEE)

^*^P and *P*1 values: significant ≤ 0.05

**Table 4:** Primary and secondary end-points of studied groups

**End-points** (*n*, %)		**At 6 m**				**At 12 m**			
		**No activity**	**Mild activity**	**Moderate activity**	**Disease flaring**	**No activity**	**Mild activity**	**Moderate activity**	**Disease flaring**
**1. Disease activity (SLEDAI)**	**HCQ**	19 (63%)	5 (17%)	4 (13%)	2 (6.7%)	21 (70%)	6 (20%)	2 (6.7%)	1 (3.3%)
	**Placebo**	17 (57%)	3 (10%)	5 (17%)	5 (17%)	15 (50%)	5 (17%)	4 (13%)	6 (20%)
	**P**	**0.178**				0.001*			
**2. LN activity**		**Partial remission**	**Complete remission**	**No remission**	**Relapse**	**Partial remission**	**Complete remission**	**No remission**	**relapse**
	**HCQ**	24 (80%)	5 (17%)	1 (3.3%)	0	11 (37%)	18 (60%)	0 (0%)	1 (3.3%)
	**Placebo**	20 (67%)	3 (10%)	5 (17%)	2 (6.7%)	13 (43%)	12 (40%)	1 (3.3%)	4 (13%)
	**P**	0.003*				0.002*			
**3. HCQ side effects**									
**Mucocutaneous**:		**No**	**Alopecia**	**Hyperpigmentation**		**No**	**Alopecia**	**Hyperpigmentation**	
	**HCQ**	30 (100%)	0	0		26 (87%)	1 (3.3%)	3 (10%)	
	**Placebo**	30 (100%)	0	0		28 (93%)	2 (6.7%)	0 (0%)	
	**P**	**1**				**0.178**			
**Fundus:**		**Normal fundus**		**Mild changes**		**Normal fundus**		**Mild changes**	
	**HCQ**	29 (97%)		1 (3.3%)		28 (93%)		2 (6.7%)	
	**Placebo**	30 (100%)		0		30 (100%)		0	
	**P**	0.933				0.36			
**Visual Acuity**:	**HCQ**	6.9 ± 1.2				7.2 ± 1.1			
	**Placebo**	7.2 ± 1.9				7.2 ± 1.2			
	**P**	**0.25**				**0.933**

**Table 5:** Multivariable Cox regression analysis of HCQ effects in studied patients

	**Regression analysis**	
	**OR (95% CI)**	***P***
**Antids-DNA**	0.428 (0.274–0.865)	0.025*
**C 3**	1.854 (0.824–2.637)	0.106
**C 4**	1.237 (0.746–3.524)	0.214
**TG**	0.724 (0.534–0.924)	0.041*
**Cholesterol**	0.634 (0.413–1.627)	0.139
**Albumin**	1.320 (0.528–4.524)	0.210
**BUN**	0.584 (0.356–2.521)	0.174
**Creatinine**	0.743 (0.415–2.149)	0.198
**24 h proteinuria**	0.423 (0.108–0.851)	0.017*
**SLEDAI**	0.352 (0.173–0.453)	0.001*
**Alopecia**	0.742 (0.427–2.541)	0.243
**Hyperpigmentation**	2.104 (0.854–4.521)	0.219
**Fundus examination**	1.684 (0.740–3.524)	0.109

*OR* odds ratio, *CI* confidence interval

^*^P value: significant ≤ 0.05

## Discussion

LN is defined as histopathologically proven glomerular immune complex deposition which activates complement, Toll-like receptors, and other inflammatory mediators leading to renal injury [12]. Treatment decisions are guided by the histological appearances that are graded according to the 2018 ISN/RPS classification [8]. Most histologically proven diseases are LN class IV, the most active disease class associated with the poorest kidney prognosis. Histological activity and chronicity characteristics can predict renal outcomes [13]. Updated treatment regimens have improved the renal and overall survival of c-SLE patients [14]. Due to its immunomodulatory, antihyperlipidemic, and antithrombotic properties, HCQ remains the main line of SLE treatment [15, 16]. The present study demonstrated that 54% of patients had LN class III, 43% had LN class IV, and 3% had LN classes IV-V. Levy and Kamphuis [17], Hiraki et al. [18], and Pereira et al. [13] also demonstrated that LN classes III and IV are the most common forms of LN in the pediatric population. The present study showed a significant reduction in serum triglycerides, cholesterol, 24-h proteinuria, and Antids-DNA levels in HCQ group after 12 months of treatment. Tam et al. [19] and Hodis et al. [20] also found significant differences in serum lipid parameters between HCQ-treated and untreated patients. Tam et al. [19], Borba et al. [21], Rahman et al. [22], and Willis et al. [23] analyzed the effect of HCQ on the lipid profile in SLE patients taking corticosteroids; they found a significant reduction in TG and cholesterol levels. In the present study, there was a significant reduction in SLEDAI scores after 6 and 12 months in the HCQ group. Mok et al. [24] reported that the prescribed HCQ dose also correlated significantly with baseline SLEDAI scores, indicating that higher doses were used for more active manifestations with serologic and clinical remission, and having therapeutic HCQ levels, a trend of lower disease activity and fewer incidences of flares were observed. Regarding renal survival, the probabilities of developing 1ry and 2ry end-points were higher in the HCQ group at 6 and 12 months as shown by Andrade Balbi et al. [25], Lee et al. [26], and Pons-Estel et al. [27] who revealed higher recovery of renal function and lower probability of kidney failure after 6 months of HCQ treatment. In addition, HCQ can delay the development of renal damage in LN with lower disease activity and glucocorticoid doses than in patients who did not receive HCQ [27]. Moreover, in the study of Ruiz-Irastorza et al. [28] and Alarcón et al. [29], chloroquine and HCQ exert a protective effect on survival, and patients treated with either of these compounds had a higher survival rate than those who were not treated with either agent. The present study demonstrated that HCQ complications were mucocutaneous (alopecia 3.3%, 10% hyperpigmentation) and ophthalmological (7% mild retinal changes without visual acuity changes). Kim et al. [30] found a retinal toxicity rate of 13.8% among patients with a 10-year mean duration of HCQ use and a mean dose of 6.4 mg/kg. Only one patient had retinal toxicity with a daily dose of < 5 mg/kg. Additionally, Melles and Marmor [31] demonstrated that 7.5% of their studied patients who had used HCQ for at least 5 years had retinal toxicity. Longer duration of HCQ use, higher daily HCQ doses, and the presence of kidney disease are factors that increase the risk of HCQ-induced retinal toxicity [30, 31].

Study limitations were the small sample size, short flow up duration, and non-monitoring the cumulative doses of HCQ and its serum levels.

## Conclusions

HCQ is an adjunctive treatment for proliferative LN that has been demonstrated to improve kidney outcomes with few side effects, such as skin hyperpigmentation and retinopathy. In addition, annual ophthalmological screening is recommended.

## Supplementary Information

Below is the link to the electronic supplementary material.

Supplementary file1 (DOCX 23 KB)

## Data Availability

The datasets used and analyzed during the current study are available from the corresponding author on reasonable request.

## Abbreviations

AF: Auto-fluorescence
anti-dsDNA: Anti-double-stranded DNA
CR: Complete remission
c-SLE: Childhood systemic lupus erythematosus
ESKD: End stage kidney disease
HCQ: Hydroxychloroquine
ISN/RPS: International Society of Nephrology/ Renal Pathology Society
LN: Lupus nephritis
MMF: Mycophenolate mofetil
OCT: Optical coherence tomography
PCR: Protein/creatinine ratio
PR: Partial remission
SLEDAI: SLE disease activity index
TG: Triglycerides
