# Supplementary material for: Hydroxychloroquine in children with proliferative lupus nephritis: a randomized clinical trial
Source: Eur J Pediatr. 2023 Feb 8;182(4):1685–95. doi: 10.1007/s00431-023-04837-0 (PMC10167107; doi:10.1007/s00431-023-04837-0)
Supplement: Supplementary file 1 — Supplementary file1 (DOCX 23 KB) [file 431_2023_4837_MOESM1_ESM.docx]

**Comparison between cases who completed the study and those who were lost at follow-up:**

| **Data** | | **All randomized cases**  **(n: 76)** | | **P** |
| --- | --- | --- | --- | --- |
|  |  | **Cases completed the study**  **(n: 60)** | **Lost cases at follow-up**  **(n: 16)** |  |
|  |  | Adherent to treatment and followed-up regularly | Non-adherent to treatment and didn’t follow-up regularly |  |
| **Age (years)** (Mean ± SD) | | 13.3 ± 2.2 | 13.7 ± 2.5 | 0.561 |
| **Sex** (n %) | Male | 8 (13%) | 3 (19%) | 0.167 |
|  | Female | 52 (87%) | 13 (81%) |  |
|  | M / F ratio | 1: 6.5 | 1: 4.3 |  |
| **Family history of rheumatological diseases** (n %) | | 7 (12%) | 0 | 0.001^*^ |
| **LN classes:** (n %)   - LN III - LN IV - LN IV-V | | 32 (54%)  26 (43%)  2 (3%) | 11 (69%)  5 )31%)  0 | 0.275 |
| **SLEDAI** (Mean ± SD) | | 24.7 ± 3.2 | 23.4 ± 4.2 | 0.733 |
